# Immune-Related Transcriptional Responses to Parasitic Infection in a Naturally Inbred Fish: Roles of Genotype and Individual Variation

**DOI:** 10.1093/gbe/evx274

**Published:** 2018-01-11

**Authors:** Rebecca Jane Pawluk, Tamsyn M Uren Webster, Joanne Cable, Carlos Garcia de Leaniz, Sofia Consuegra

**Affiliations:** 1 Department of Biosciences, College of Science, Swansea University, Singleton Park, Wales, United Kingdom; 2 Cardiff University, School of Biosciences, Wales, United Kingdom

**Keywords:** *Kryptolebias marmoratus*, transcriptome, *Argulus foliaceus*, immune response, inbreeding

## Abstract

Parasites are strong drivers of evolutionary change and the genetic variation of both host and parasite populations can co-evolve as a function of parasite virulence and host resistance. The role of transcriptome variation in specific interactions between host and parasite genotypes has been less studied and can be confounded by differences in genetic variation. We employed two naturally inbred lines of a self-fertilizing fish to estimate the role of host genotype in the transcriptome response to parasite infection using RNA-seq. In addition, we targeted several differentially expressed immune-related genes to further investigate the relative role of individual variation in the immune response using RT-qPCR, taking advantage of the genomic uniformity of the self-fertilizing lines. We found significant differences in gene expression between lines in response to infection both in the transcriptome and in individual gene RT-qPCR analyses. Individual RT-qPCR analyses of gene expression identified significant variance differences between lines for six genes but only for three genes between infected and control fish. Our results indicate that although the genetic background plays an important role in the transcriptome response to parasites, it cannot fully explain individual differences within genetically homogeneous lines, which can be important for determining the response to parasites.

## Introduction

Parasites are one of the strongest drivers of evolutionary change, influencing the abundance, distribution and genetic variability of their hosts (Ebert and Hamilton 1996). The genetic composition of both parasites and hosts can change as a result of co-evolution between parasite virulence and host immune resistance (Hamilton 1980). In this sense, the Red Queen Hypothesis predicts that parasite-mediated selection against hosts with common genotypes should help maintain high genetic variability in the offspring (Morran et al. 2011), thereby providing a potential explanation for the maintenance of sexual reproduction despite the costs of producing males (Howard and Lively 1994; Morran et al. 2011). Since the immune response against parasites involves costs and life-history trade-offs (Sheldon and Verhulst 1996; Lochmiller and Deerenberg 2000), hosts need to optimize their defence mechanisms, for example by developing long-term immunity only to certain parasites (e.g., those with high transmission rates and intermediate virulence; Boots and Bowers 2004). Parasites, in turn, can develop local adaptations to the hosts’ most common genotypes, exhibiting greater fitness in local than in allopatric hosts (Greischar and Koskella 2007). The extent to which parasites can be locally adapted will largely depend on migration rates exhibited by both hosts and parasites (Gandon et al. 1996), their genetic background (Andrade et al. 2002), but critically, also on the interactions between host and parasite genomes (Lambrechts et al. 2006).

Environmental conditions influence the presence and abundance of hosts and their parasites, thereby shaping the composition of parasite communities (Evans and Neff 2009; Eizaguirre and Lenz 2010). However, host resistance seems to be influenced by only a few loci and their epistatic interactions and this may depend more on the genotype combination of hosts and parasites than on the environment (Wilfert and Schmid-Hempel 2008). The host genotype can modulate parasite fitness, infection patterns, and virulence by influencing the strength and direction of selection on parasite evolution (De Roode and Altizer 2010). For example, in the snail *Biomphalaria glabrata*, genetic background can influence responses to infection by the castrating parasite *Schistosoma mansoni* as well as the parasite’s reproductive success (Tavalire et al. 2016). In Chagas disease, which has a variable spectrum of pathology caused by *Trypanosoma cruzi*, parasite distribution between tissues depends in part on the genetic makeup of the parasite, but largely differs depending on the host’s genotype (Campbell et al. 2004), specifically on host’s loci involved in the modulation of the infection (e.g., the genes of the Major Histocompatibility Complex or MHC—Andrade et al. 2002).

Genotype-by-genotype interaction between hosts and parasites can result in increasing host genetic diversity, for example, allelic diversity in immune-related genes (Carius et al. 2001). The highly polymorphic MHC genes are probably the most studied in host–parasite genotype interactions (Bernatchez and Landry 2003). However, the MHC-based adaptive immune response can be a relatively slow response to infection (Acevedo-Whitehouse and Cunningham 2006), and it does not fully explain host resistance (Rauch et al. 2006b). Growing evidence suggests that the innate immune response plays a central role in the interactions between host and parasite genotypes, as it has a tight relationship with acquired immunity (Magnadóttir 2006) and provides a more immediate form of defence against pathogens, which could act within hours of infection (Rauch et al. 2006a).

Specific interactions between host and parasite genotypes also manifest as variation in gene expression when, for example, different parasite genotypes elicit variable responses in the host transcriptome (Barribeau et al. 2014). Differences in immune gene expression have been observed in response to infection by different parasite genotypes in several organisms, including bumble bees (Barribeau et al. 2014) and sticklebacks (Haase et al. 2014). Host-specific gene expression responses to parasite infection can be more challenging to identify, particularly in vertebrates where individual genetic variability confounds interpretation. Laboratory inbred mice lines, along with other model organisms, are commonly used to help understand the role of host genetic background in response to infection (Srivastava et al. 2009) and the influence of allele-specific variation on transcript abundance (Keane et al. 2011). However, the extent to which results from model organisms can explain processes ocurring in natural populations is debatable, and it is recognized that evolutionary mutant models (organisms with particular traits of interest that have developed over generations of natural, instead of artificial, selection) can provide additional insights into the genetic factors and gene-by-environment interactions that affect the immune response (Albertson et al. 2009). With this in mind, we took advantage of the self-fertilizing and naturally inbred mangrove killifish (*Kryptolebias marmoratus*) to examine the relative roles of genotype versus individual variation in the immune-related transcriptome response of the host to parasite infection.

## Materials and Methods

### Study Species and Experimental Design

We used two different *K. marmoratus* selfing lines (R and DAN) originating from Belize that have undergone at least 30 generations of self-fertilization (Ellison et al. 2013). Fish were reared in individual aquaria (12×8×8.5 cm^3^) containing 750 ml of brackish water (14 ppt salinity, constituted from dechlorinated water and marine-filtered water) under controlled conditions (12:12 h light:dark photoperiod, 24 °C). Eighty 8-month-old *K. marmoratus* were selected from the two lines (40 DAN and 40 R). Twenty fish from each line were size-matched and were individually infected with a single *Argulus foliaceus* (an ectoparasitic freshwater louse) following Stewart et al. (2017), whereas the other 20 from each line were unexposed to the parasite to serve as controls. The culture of *A. foliaceus* originated from carp (*Cyprinus carpio*) caught in a still water fishery in North Lincolnshire, July 2014, and thereafter was maintained on *Gasterosteus aculeatus* (three-spined sticklebacks) at Cardiff University as detailed in Stewart et al. (2017). *A. foliaceus* is a generalist parasite that tends to spend variable periods away from the host, often resting on the substrate and can be monitored visually without anesthetizing the fish. The attachment of the parasites was facilitated by manually placing a single *Argulus* on each fish’s skin, allowing for suction by the parasite to the body surface and afterwards the presence of the parasite attached to the fish was visually monitored every 2 h. Fish size was not significantly different between lines (DAN mean size = 14.8 mm, SD = 0.027; R mean size = 14.3 mm, SD = 0.012; Mann–Whitney U = 287.000; *P *=* *0.984) and *Argulus* successfully attached to all exposed fish, with attachment times varying between 18 and 48 h. Attachment times (grouped by 6 h intervals) did not differ between lines (Kruskal–Wallis chi-squared = 1.52 df = 4, P = 0.822). After 48 h approximately 50% of the fish were still infected and the experiment was terminated, all fish were humanely euthanized, stored in RNA later and frozen at −80°C prior to RNA extraction and library preparation.

#### RNA Extraction, Library Construction and Sequencing

For transcriptomic analyses, we selected fish that had remained infected for 48 h. Total RNA was extracted from 20 whole individuals (5 R controls, 5 DAN controls, 5 R infected individuals, 5 DAN infected individuals) using the Bioline Isolate II RNA mini kit (Bioline, London, United Kingdom) according to the manufacturer’s instructions. The concentration and quality of RNA in each sample was determined using a NanoDrop 2000 Spectrophotometer (NanoDrop Technologies, USA) and a Qubit fluorometer. Library construction was completed using the Illumina TruSeq kit v2 according to the manufacturer’s instructions (Illumina, San Diego, CA) using 500-1,000 ng of RNA. The concentration and quality of the libraries were determined using the Qubit (Invitrogen) and Bioanalyzer 2100 (Agilent Technologies). All samples were sequenced (126 bp paired end) on an Illumina HiSeq2500 platform (Illumina, San Diego, CA, USA). Samples were pooled for library construction when RNA concentrations were <500 ng resulting in four pool groups of three fish (control DAN, control R, infected DAN, infected R), one pool of two fish (infected R) and six fish individually sequenced (two of each control DAN, control R, infected DAN).

#### Transcriptome Assembly and Annotation

Raw sequences were processed using Trimmomatic, version 0.33 (Bolger et al. 2014), to ensure Illumina adapter sequences were removed and poor quality 3′ ends were trimmed using a sliding window (*Q* > 20). De novo transcriptome assembly was conducted with reads from all combined samples using Trinity version r2013-02-25 (Grabherr et al. 2011; Haas et al. 2013), encompassing an initial in silico normalization with an optimized *K* value of 40. The final transcriptome assembly was annotated using Blastx (Altschul et al. 1990) against Ensembl peptide databases for zebrafish, medaka, stickleback, mice, and humans using an *e*-value cutoff < 1e−5. Most of the annotations were from zebrafish. Additional annotation of the remaining unidentified sequences was conducted using Blastn against NCBI refseq databases in order to obtain as many annotated sequences as possible.

#### Differential Gene Expression Analysis and Functional Analysis

In addition to the samples that were sequenced as pools due to low RNA concentration, three sequence pools were made from the individually sequenced samples (two control fish DAN, two infected fish DAN, and two control fish R) to ensure a balanced number of pooled samples per treatment (i.e., two pools for each one of the DAN and R infected and control groups, each pool consisting of two and three individuals, respectively). As the number of reads were similar between pairs of individuals to be pooled (Supplementary material table S1), we randomly down-sampled the sequence reads of the largest library of each pair to the size of the smallest one, using the functions cut (to downsize) and cat (to pool). Bowtie2 v.2.0.2 (Langmead and Salzberg 2012) was used to align reads from all pools against the final transcriptome assembly, using the K 1 parameter to report a single best hit for each read. Following alignment, read counts for each transcript were generated using idxstats in Samtools v.1.2, (Li et al. 2009).

The EdgeR package (Robinson et al. 2010) was used to calculate significant differences in gene expression between infected and non-infected groups of fish from the two different selfing lines (DAN and R) using treatment and line as factors, via a quasi-likelihood negative binomial generalized log-linear model (glmQLFTest). Only transcripts with >4 reads were considered and prior to the analysis tagwise dispersion was used to moderate the degree of over-dispersion amongst transcripts using the recommended prior, df, of 10. Transcripts with FDR < 0.05 and *P* < 0.001 were considered to be differentially expressed (DE). In addition, a Gene ontology (GO) enrichment analysis was completed for DE transcripts, using the Database for Annotation, Visualization and Integrated Discovery, version 6.8 (DAVID) (Dennis et al. 2003).

#### RT-qPCR Analysis

To further assess the role of individual variation versus genetic background in the gene expression in response to infection, we selected a group of immune-related genes from the transcriptome analyses which were analyzed using real-time quantitative PCR (RT-qPCR) in 48 fish (24 DAN and 24 R, including 9 of the fish sequenced for RNA-seq, of which there were 9 controls and 15 infected fish from each line). Four immune-related genes were selected among those DE (*MHC I-uka*, *MHC II-dab*, *CD4-1*, and *CXCL 11.8*; FDR < 0.05 and *P* < 0.0001) and five more based on *P* < 0.05 (*LECT2*, *C7*) or fold change >2 (*AHSA1B*, *FGG* and *IRGF1*) (Supplementary material S1, tables S2 and S3). *18S rRNA* and *EF1a* were used as reference genes following previous work (Olsvik et al. 2005; Small et al. 2008; Rhee et al. 2010). Specific primers for immune targets were designed using NCBI primer-BLAST (Ye et al. 2012), followed by Beacon designer (ver. 2.1, PREMIER Biosoft) to check for the absence of secondary structure. For this analysis, RNA from 48 fish (15 infected fish from each line and 9 control fish from each line) was extracted as described earlier. The concentration and purity of RNA in each sample was determined using a Qubit fluorometer and a NanoDrop 2000 Spectrophotometer. Total RNA (2 µg) was first treated with DNase (Promega), then reverse transcribed with GoScript Reverse Transcriptase (Promega, Medison, Wisconsin) using 10 µM random hexamer primers (MWG-Biotech).

All primer optimization and amplification reactions were completed using 5 µl SYBR green Supermix (Biorad), 3.5 µl water, 0.25 µl forward primer (10 µM), 0.25 µl reverse primer (10 µM) and 1 µl cDNA per sample. A CFX96 Touch Real-Time PCR Detection System (Biorad) was used to run samples using the following protocol: 95 °C for 10 min; 40 cycles of 95 °C for 10 s, 60 °C for 45 s; 95 °C for 1 min; 55 °C for 1 min; 80 cycles starting at 55 °C for 10 s with a melting curve program of 55–95 °C and a heating rate of 0.5 °C every 10 s. The annealing temperature was adjusted accordingly to optimize primer efficiency for each target gene (Supplementary material S1, table S4). PCR efficiencies (*E* = 10^[^^−^^1/slope]^) for each primer pair were derived from standard curves (mean quantification cycle (Cq) vs. log cDNA dilution) using a 2 or 10-fold dilution series with pooled cDNA. All optimized primer pairs had efficiency values between 89.5 and 119.2, and standard curve *R*^2^ values > 0.95 (Supplementary table S4). Melt curve analysis confirmed the specific amplification of a single PCR products in each case. Following primer optimization all samples (diluted at 1:2) were run in triplicate for each gene in accordance with the sample maximization method (Hellemans et al. 2007). Samples displaying non-specific amplification (contaminant melt peaks) or high variation in Cq values between technical replicates (standard deviation—SD—>1.0) were removed from the analysis.

BestKeeper (Pfaffl et al. 2004) was used to estimate the stability of the reference genes. Mean Cq values were extracted for all samples and the relative expression of each gene was calculated using the comparative 2^−^^ΔΔC^^*t*^ method including gene-specific efficiency correction (Pfaffl 2001), and normalizing to the geometric mean for reference genes (Vandesompele et al. 2002; Hellemans et al. 2007).

#### Data Analysis

Differences in individual gene expression between groups (fish from different lines and treatment) were initially assessed with a generalized linear model using the function *glm* in R version 3.4.0 and a Gaussian link function. The full model included infection status (yes/no) and genetic background (line) as fixed factors plus their interactions. Model selection was carried out based on AIC (Akaike Information Criterion) and log-likelihood (LR) ratio analyses. Generalized Linear Mixed-effects models (GLMM) of the combined gene expression for the target genes were fitted using the *lmer* function in the R package *lme4* (Bates et al. 2014) using individuals as random effects. Comparisons between models with and without random factors were carried by AIC comparisons with respect to the GLMM fitted by Maximum Likelihood. Variances between groups were compared using the Fligner–Killeen test of homogeneity of variances (Conover et al. 1981). All analyses were run on R version 3.4.0 (R Core Team 2014).

## Results

### Transcriptome Comparison between Treatments

**Fig. 1.**
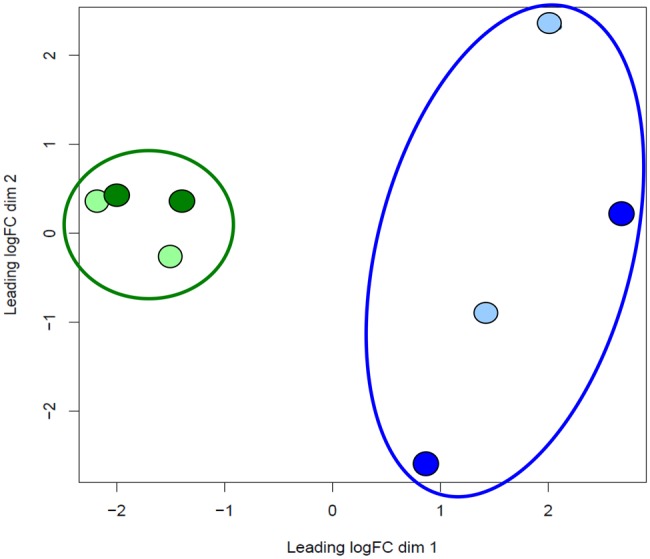
—Multidimensional scaling (MDS) plot of transcriptome analyses of four treatment groups of *K. marmoratus* (R control: Light green, R infected: dark green, DAN control: light blue, DAN infected: dark blue).

We assembled a de novo transcriptome of *K. marmoratus* from 294 million Illumina RNA-sequencing reads derived from the 20 fish from two different inbred lines (DAN and R) subject to two treatments (infection with *A. foliaceus* and non-infected control). The final assembly consisted of 291,771 transcripts with an average length of 1,136 bp and a N50 of 2,575 bp. Annotation against Ensemble databases resulted in a total of 67,822 annotated transcripts. EdgeR analysis identified 276 DE genes between groups (FDR < 0.05; *P* < 0.001); (Supplementary material S1, table S3). Most of the differences were due to line (205 genes were DE when only differences between lines were considered). MDS grouping and a heat map indicated greater differentiation between lines than between treatments (fig. 1 and Supplementary material S2, fig. S1). The DE genes included several immune-related genes (*C7, MHC I-uka, MHC II-dab, CD4-1*) as well as genes involved in response to bacteria (*LECT2*) and inflammation (*CXCL11.8*).

#### Functional Analysis of Treatment Group Differences

Functional analysis of DE genes revealed 23 enriched GO terms (*P *<* *0.1) (Supplementary material S1, fig. S2) and six annotation clusters, 13 of which were involved in immune response (Level: GO ALL). In addition to genes directly involved in immune response, many genes involved in membrane transfer and GTPase activity were highly enriched. Functional classification confirmed that the major enriched gene families included the Major Histocompatibility Complex and Immunoglobulin families (Supplementary material S1, tables S5 and S6).

#### Individual Gene Analyses

**Fig. 2.**
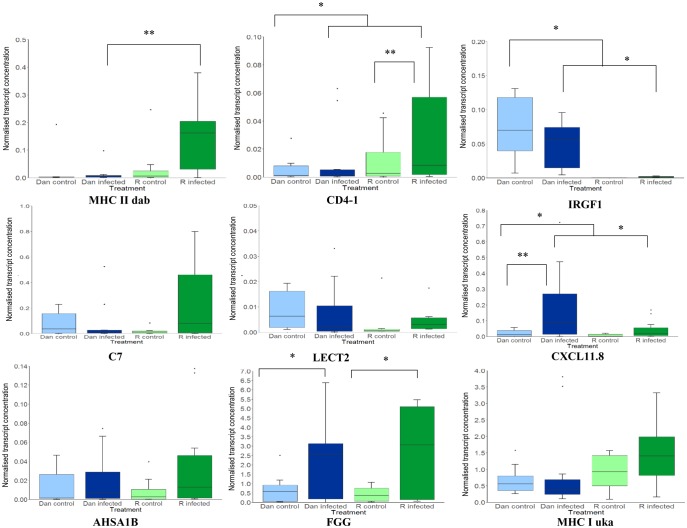
—Relative differences in expression (calculated using the comparative 2^−ΔΔC^^*t*^ method and analyzed using a Generalized Linear Mixed-effects model) of nine target immune-related genes. Four groups were analyzed using RT-qPCR: two lines—R (green) and DAN (blue), as well as two treatments—infected (dark) and control (light). Asterisks indicate significance *P<0.5, **P<0.01.

The stability of the reference genes was estimated by their SD and the correlation with the BestKeeper index. SD was 1.51 for *18 S rRNA* and 1.50 for *EF1a*, both were highly correlated (Pearson *r* = 0.944, *P* = 0.001) and displayed high correlation with the BestKeeper index (*18 S rRNA*: *r* = 0.986, *P* = 0.001; *EF1a*: *r* = 0.986, *P* = 0.001). Samples were tested for their expression stability using the intrinsic variance of expression (InVar) implemented in BestKeeper and five of them (two DAN and three  R) were excluded from the rest of the analyses based on their high (>3) overexpression values, as recommended in Pfaffl et al. (2004). Of the nine immune-related genes analyzed individually using RT-qPCR (*MHC I-uka, MHC II-dab, FGG, IRGF1, C7, CXCL11.8, CD4-1, LECT2*, and *AHSA1B*), five displayed significant differences either in expression between lines (*MHC II-dab*, *P* = 0.013; *IRFG1*, *P* = 0.037), infection status (*FGG*, *P* = 0.034), or both (*CD4-1 *line, *P* = 0.048, infection *P* = 0.011; *CXCL11.8*, line *P* = 0.0025, infection *P* = 0.016) (fig. 2). There were no significant interactions between the genetic background (line) and infection status in any case. The role of the individual immune response versus the genetic background (line) was estimated by analyzing the expression of all the nine target genes and of the five DE genes in relation to infection and line. Comparison among four models including and excluding interactions between line and infection, two of them including individual (ID) as random factor, indicated that the model which included infection, line and ID (but no interactions) provided the best fit to the data (lmer(Gexpress∼Line + Infect + (1ID))) when all the nine candidate genes were considered (AIC = 1045.6; *χ*^2^ = 3.8410, df = 1, *P* = 0.05), although considering ID as random factor did not improve the fit over the most basic model (lmer(Gexpress∼Line + Infect)) when only the five DE genes were considered (supplementary material S2, fig. S5, Supplementary Material online).

Six of nine genes (*IRGF1, CD4-1, MHC II-dab, AHSA1b, CXCL11.8*, and *LECT2*) displayed significant differences in variance between lines (supplementary material S2, fig. S3, Supplementary Material online). Of these, *MHC II-dab* and *CXCL11.8* additionally displayed significant differences in variance between treatments (infected vs. control). In addition, *FGG* displayed significant differences only between treatments (table 1). For all nine genes, the variance was higher within infected than control groups.

**Table 1:** Variance (Var) in Gene Expression Compared Between the Two Lines of *K. marmoratus* (R and DAN) and Treatments (Infection and Control) for Each One of Nine Immune-Related Target Genes, Analyzed Using RT-qPCR

	*MHC I*	*IRGF1*	*CD4-1*	*FGG*	*C7*	*MHC II*	*AHSAb1*	*CXCL11.8*	*LECT2*
Var line DAN	0.9531	0.1729	0.0003	2.6146	0.0158	0.0019	0.0005	0.0337	0.0007
Var line R	0.7725	0.0000	0.0012	3.9938	0.0914	0.0132	0.0016	0.0023	0.0000
*P* (between lines)	0.072	*3.321e−07*	*0.012*	0.627	0.057	*8.61e−05*	*0.038*	*0.018*	*0.008*
Var Control	0.2302	0.0418	0.0002	0.4798	0.0052	0.0059	0.0002	0.0003	0.0001
Var Infection	1.2466	0.1306	0.0010	4.2703	0.0755	0.0095	0.0014	0.0279	0.0006
*P* (between treatments)	0.096	0.109	0.154	*0.011*	0.102	*0.038*	0.769	*0.024*	0.447

Note.—Significant differences in italics (*P* < 0.05). The target genes chosen serve important immune functions including antigen presentation (*MHC I-uka, MHC II-dab, CD4-1*), membrane regulation and attack (C7 and *IRGF1*), clot formation (*FGG*), immune regulation and cell trafficking (*LECT2* and *CXCL11.8*) and the ATPase activity of heat shock proteins (*AHSAb1*). Variances between groups were compared using the Fligner-Killeen test of homogeneity of variances (Conover et al. 1981).

## Discussion

Evidence of the role of genotype-specific resistance and infectivity in maintaining the dynamics of the host–parasite arms race has been accumulating for both plants and animals over the last decades (Carius et al. 2001; Srivastava et al. 2009; Rodenburg et al. 2017). However, the importance of transcriptome variation in response to specific interactions between host and parasite genotypes has only received attention recently (Barribeau et al. 2014). Specific transcriptome responses to parasite infection can be difficult to identify in natural populations where there is high individual genetic variability. By using naturally inbred (selfed) fish reared under controlled conditions, we have been able to estimate significant differences in transcript expression between genotypes, as well as variance differences in gene expression which were significant between lines and treatments.

The parasite we used (an argulid) induces a consistent innate response with the addition of an adaptive response 7–10 days’ post-infection (Stewart et al. 2017). We found a number of DE genes, with higher levels of differentiation between selfing lines than between treatments (infected and controls fish). In addition, we examined several immune-related genes that displayed differential expression between lines and/or infection status, selected based on FDR < 0.05 and *P* < 0.05. In addition, as we had no a priori assumption of the number of DE genes that we would find in relation to infection and/or line differences, we used a threshold of fold change >2, which is often considered as a significant change in the expression of immune related genes in fish in response to infection (e.g., Purcell et al. 2004; Polinski et al. 2014; El Aamri et al. 2015), as the limit below which we were not further investigating a particular gene. Five of the nine genes selected on this basis were found DE in the qPCR analyses either between lines, treatments or both. Of them, *CXCL11.8*, a chemokine involved in regulating cell trafficking of leukocytes that can be critical for the recruitment of immune cells to the sites of infection, has a dual role in immune response and normal physiological conditions (Alejo and Tafalla 2011). It was targeted for qPCR analysis based on its differential expression between lines (upregulated in DAN) and the individual analyses indicated that differential expression between control and infected groups and between lines, as well as higher level of individual variation among DAN than R individuals, supporting the results from the transcriptome. *MHC* class II-dab was also DE between lines (upregulated in R) based on EdgeR analyses, and this was confirmed on the individual qPCR analysis, which also indicated differences in variance between line and treatment groups. *MHC II* molecules are expressed on antigen presenting cells which have direct functional relevance to teleost immune responses (Janeway et al. 2004; Chen et al. 2006; Hofmann et al. 2017) and have been identified in various teleost species (Grimholt et al. 2000; Pang et al. 2013; Hofmann et al. 2017) with very high allelic diversity maintained by natural and sexual selection (Aguilar and Garza 2007; Consuegra and Garcia de Leaniz 2008), which could result in allele specific differences in gene expression. *MHC II* expression in *K. marmoratus* was consistent with that observed in Atlantic salmon when infected with lice from the genus *Lepeophtheirus* (see Fast et al. 2006). *CD4* binds to *MHC II* molecules on the surface of dendritic cells which are important for antigen presentation (Leahy 1995; Yoon et al. 2015). In contrast, and despite its high degree of polymorphism and the differential expression observed in the transcriptome analysis, *MHC class I* gene expression and variance did not differ between infected R and DAN individuals (although R fish tended to have higher expression of this gene, similar to that observed at the transcriptome level). *MHC I* is responsible for the presentation of intracellularly derived antigens to the TCR/CD8 complex of cells and, as for *MHC II*, its high polymorphism is likely to be maintained by natural and, potentially, sexual selection (Ellison et al. 2012, 2013). As for *MHC II-dab*, we found that *CD4-1* displayed differential expression between lines and between control and infected groups based on transcriptome analyses (more expressed in DAN and infected individuals). Individual qPCR confirmed differences in expression between lines and between infected and control fish, albeit in the opposite direction (overexpressed in R). The difference between analyses could be the result of individual variation, as potentially indicated by the significant differences in variance between lines.

*FGG* (one of the genes encoding the peptide chains of fibrinogen) is important for the formation of clots which can be relevant for the response to external parasites (Vo et al. 2013). Although not in the original list of DE genes, we targeted it based on having fold change >2 and in the individual analysis we found it DE between control and infected groups in both lines. Individual variation was much higher in infected individuals than in control specimens but did not differ significantly between lines. In contrast, *IRGF1* (an immunity-related GTPase) displayed high variation in amplification between lines, both at the transcriptome and qPCR levels. *IRGF1* only amplified in 22% of R control specimens and 12% of R infected specimens, with very low levels of expression in all cases and displayed significantly larger individual variance in infected R individuals than DAN.

*C7* and *LECT2* play a role in the complement activation in fish (*C7*; Guo et al. 2016) and in the immune regulation response to bacterial infection (*LECT2*; Lin et al. 2007; Chen et al. 2009; Lu et al. 2013) and were listed as DE with *P* < 0.05 in the transcriptome analysis. Both had higher number of counts in the transcriptome of DAN fish compared to R and displayed higher variance in infected R individuals when compared with DAN in the individual analyses, although the differences were not significant. No differences were observed either in the expression or variance of *AHSA1B*, involved in the regulation of cell growth and apoptosis (Shao et al. 2016), which had been selected on the basis of a fold change >2.

In general, the results of the transcriptome and the individual analyses displayed a good agreement, with five of the nine genes identified as DE in the transcriptome being confirmed at the individual level (all but one in the same direction). Comparing control and exposed fish was critical to be able to interpret the potential genetic or individually linked differences in gene expression in response to infection. Our transcriptome results indicate that the differences in gene expression were larger between selfing lines than between infected and control fish, and this pattern was supported by the targeted approach looking at selected immune-related genes, suggesting that there could be a genotype-related pattern of gene expression, similar to the one described in bumble bees (Barribeau et al. 2014). Natural variation in gene expression has also been oberved in populations of contrasting *Fundulus* species, where 18% of 900 genes displayed significant differences in expression among wild individuals within populations (Oleksiak et al. 2002). Individual patterns of gene expression in humans also display differences among individuals that can be as large as those comparing humans and chimpanzees (Enard et al. 2002). Individual variation in gene expression in human blood seems to be variable among genes, but crucially several of the genes identified as having high intrinsic variation are immune-related genes with high polymorphism (e.g., *MHC II* genes) (Enard et al. 2002), suggesting that genotype variation can be (at least in part) responsible for the differences in gene expression. Microsatellite analyses had previously indicated that fish from the R line were genetically identical and homozygous at 28 of 29 microsatellites, whereas fish from the line DAN formed three different homozygous genotypic groups separated by variation at only one microsatellite locus (Ellison et al. 2013). Both DAN and R selfing lines displayed significant differences in the degree of variance in gene expression for the nine target genes, and the variance was consistently larger among infected than among control individuals, suggesting an individual component of the immune response, despite the genetic homogeneity of the individuals from each line. Variation in the regulatory regions of the DNA that affect gene expression (e.g., transcriptional regulatory sequences) result in individual variation in expression patterns (Handunnetthi et al. 2010), which can be targets of selection and play an important role in adaptation. This could explain the differences in variance observed between lines, despite their high homozygosity and highly inbred condition. Yet, it does not fully explain the individual variation in gene expression observed within each experimental line, where individuals were genetically homogeneous, offspring the same age from the same parent and reared in a common environment, and we cannot rule out that other mechanisms, such as gene expression stochasticity, could be involved. The model that best predicted the gene expression patterns included individual as random factor, albeit only when all the nine target genes where included, suggesting that not only the genotype, but also intrinsic factors could be involved in the immune-related gene response to infection. Stochasticity in gene expression has been observed for example in isogenic bacteria subject to identical environmental conditions, resulting in phenotypic differences (Thattai and Van Oudenaarden 2004). As for stochasticity among cells from a single organism (Kaern et al. 2005), in whole individuals this could represent a mechanism which provides flexibility to survive in fluctuating environments.

Whether regulated by genotype variation or not, this study suggests that individual differences in gene expression can also be important for determining the response to parasites. Given that the response to parasitic infection cannot normally be explained by genotype alone, we suggest that the naturally inbred mangrove killifish is an ideal model species to further investigate transcriptomic responses to vertebrate infection and their regulation mechanisms.

## Supplementary Material

Supplementary data are available at *Genome Biology and Evolution* online.

## Supplementary Material

Supplementary Data
