# Supplementary material for: Immune-Related Transcriptional Responses to Parasitic Infection in a Naturally Inbred Fish: Roles of Genotype and Individual Variation
Source: Genome Biol Evol. 2018 Jan 11;10(1):319–27. doi: 10.1093/gbe/evx274 (PMC5786212; doi:10.1093/gbe/evx274)
Supplement: Supplementary Data [file evx274_supp.zip › Supplementary material 2_to submit_101117.docx]

**Supplementary material 2 for: Immune-related transcriptional responses to parasitic infection in a naturally inbred fish: roles of genotype and individual variation**

Rebecca Jane Pawluk, Tamsyn Uren-Webster, Joanne Cable, Carlos Garcia de Leaniz,

Sofia Consuegra

**Figure S1.** Heat map of differentially expressed genes (Log 10 transformed count data) between treatments (infection with *Argulus foliaceous* and control) of two inbred strains of *Kryptolebias marmoratus* (R and Dan)*.*


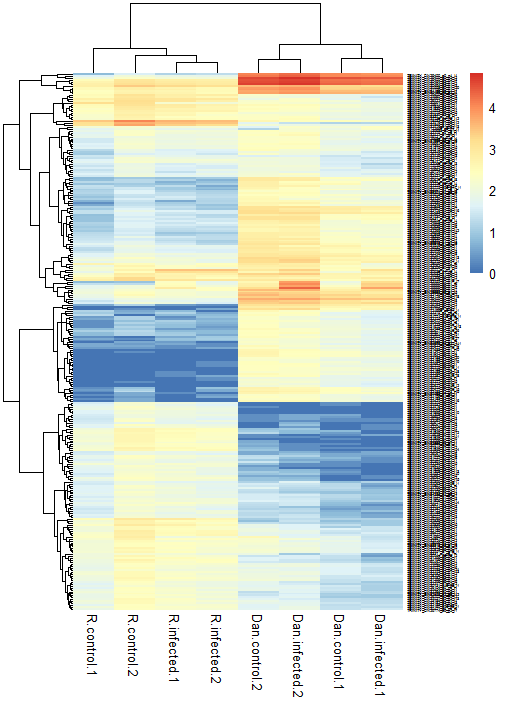


**Figure S2**. Enriched gene ontology (GO) classifications at Level: GO ALL, for all differentially expressed genes (from results of EdgeR glm analysis), P<0.1.


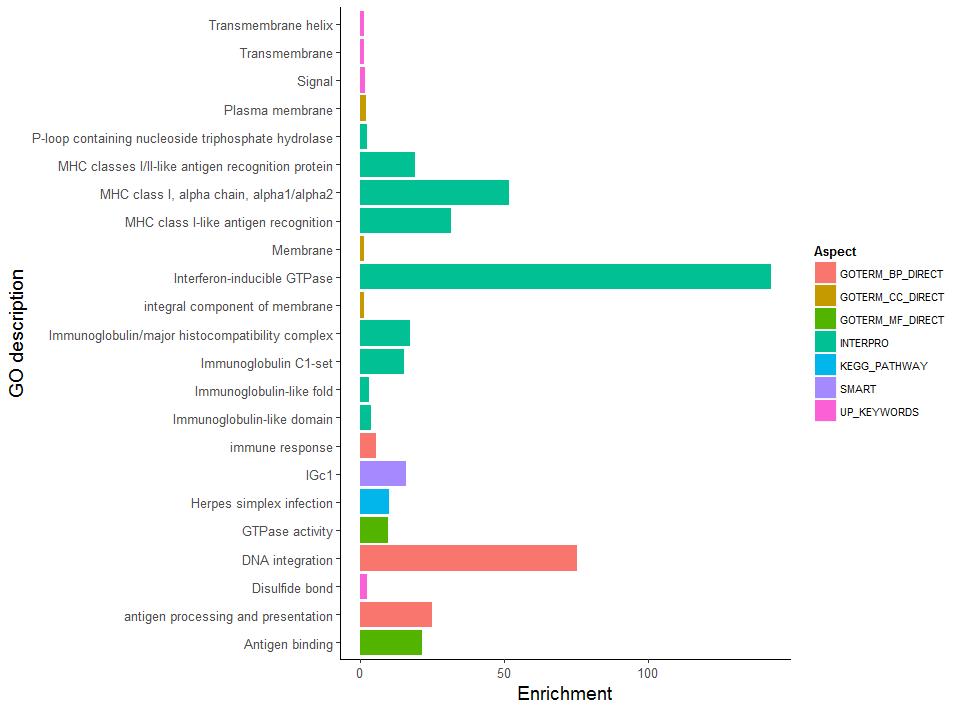


**Figure S3**. Individual Ct values for 9 target immune-related genes. Four groups were analysed using RT-qPCR: two lines- R (green) and DAN (blue), as well as two treatments- infected (dark) and control (light).


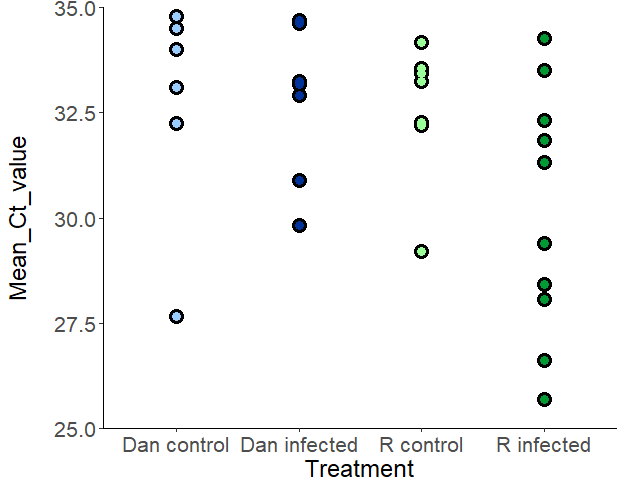

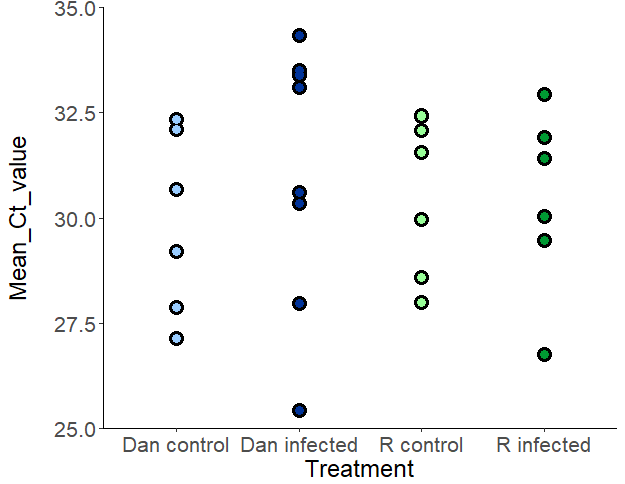

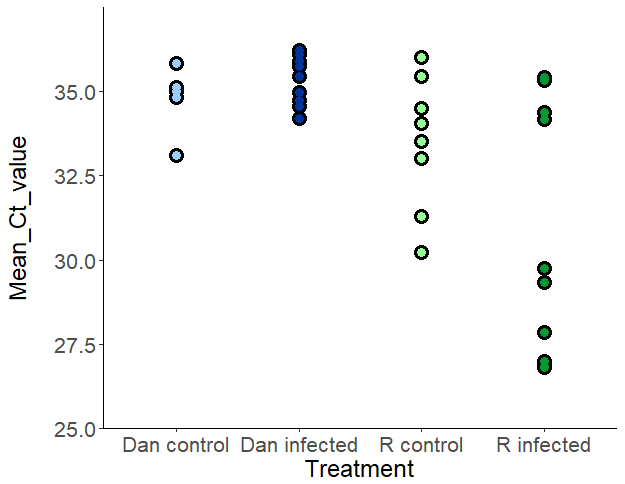

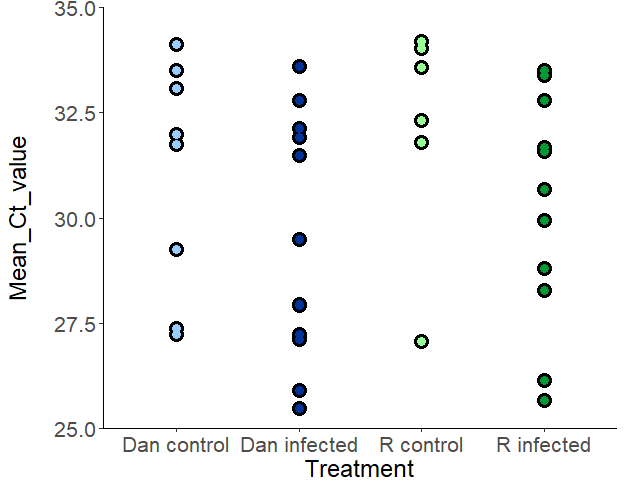

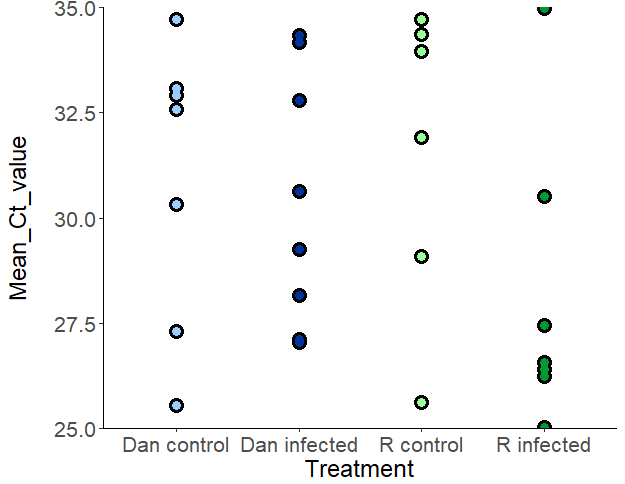

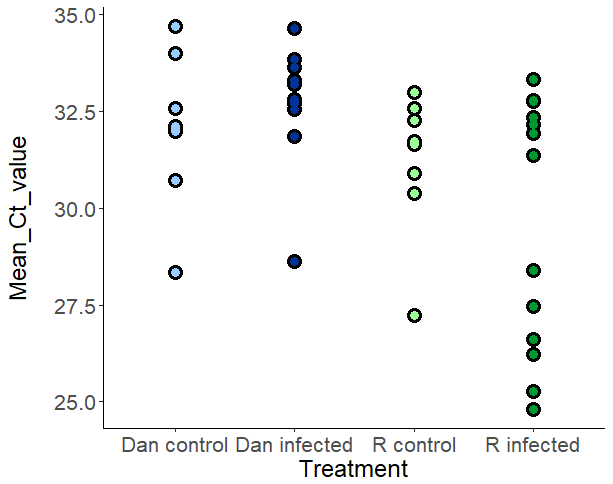


*CD4-1*


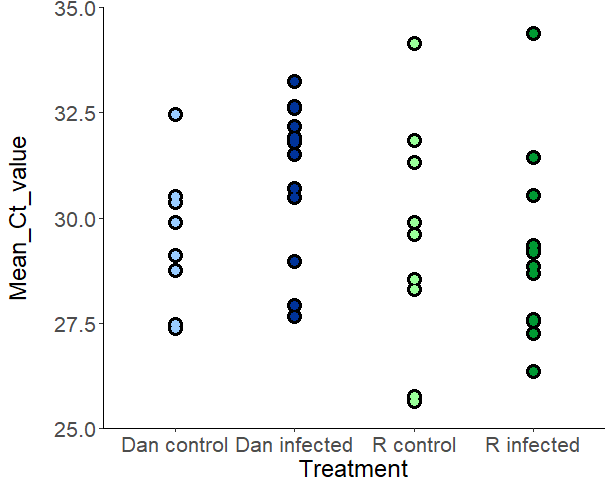


*MHC1-uka*


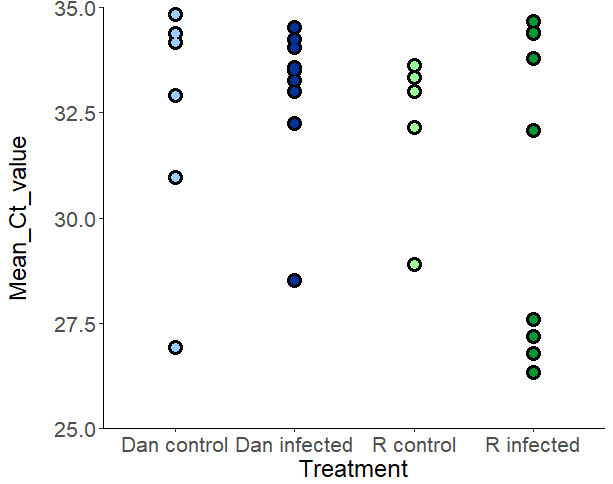


*C7*

*FGG*

*CXCL11.8*

*MHC2-dab*

*LECT2*

*AHSA1B*


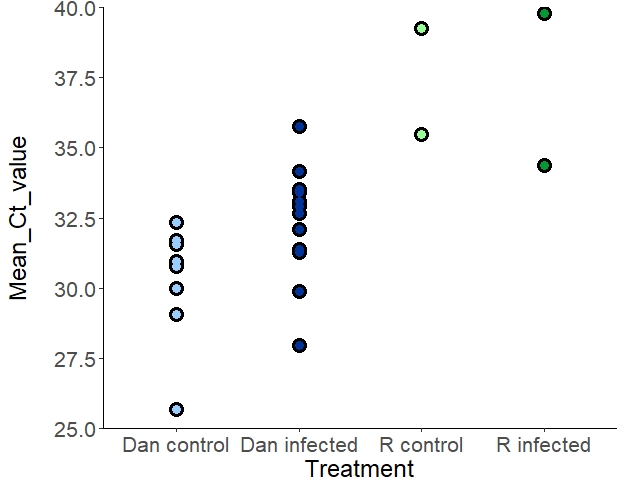


*IRGF1*

**Figure S4.** Generalized Linear Model with Quasi-Likelihood Test for the analysis of differential gene expression in *Kryptolebias marmoratus* as a function of treatment (infection or control) and selfing line (DAN and R).

targets <- readTargets ("label_file_24.txt")

x <-read.delim ("o_samples_input_matrix2", row.names=1, stringsAsFactors=FALSE)

head (x)

dim (x) #check no. sequences and sample columns

colSums (x) #reads per sample

colSums(x) / 1e06 #reads in millions

table (rowSums (x)) [1:30] # output no. sequences with low counts (from 0-30)

Treatment <-factor(targets$treat,levels=c("Sham","Infected"))

Strain <- factor (targets$strain, level=c("D","R"))

y = DGEList(counts=x[,1:8])

keep <- rowSums(cpm(y)>1) >= 4

y <- y[keep, , keep.lib.sizes=FALSE]

y = calcNormFactors (y)

design2 <- model.matrix(~Treatment * Strain, data=targets)

y <- estimateGLMCommonDisp(y,design2, verbose=TRUE)

y <- estimateGLMTrendedDisp(y, design2)

y <- estimateGLMTagwiseDisp(y, design2)

fit <- glmQLFit(y, design2)

colnames(design2)

[1] "(Intercept)" "TreatmentInfected" "StrainR"

[4] "TreatmentInfected:StrainR"

qlf <- glmQLFTest(fit, coef=2:4)

tab <- topTags(qlf, n=67824)

FDR <- p.adjust(qlf$table$PValue, method="BH")

is.de <- decideTestsDGE(qlf)

**Figure S5.** Comparison among models of gene expression in *Kryptolebias marmoratus* as a function of treatment (infection or control) and selfing line (DAN and R), including and excluding individual (ID) as a random factor. (a) All nine immune-related genes targeted for qPCR (*MHC1uka, MHC2dab, Fgg, IRGF1, C7, cxcl11.8, Cd4-1, Lect2 and Ahsa1b*) (b) target immune-related identified as DE in qPCR analyses (*MHC2dab, Fgg, cxcl11.8, Cd4-1, IRGF1*)

**(a)**

> m1<-lmer(Gexpress~Line+Infect+(1|ID))

> m2<-lmer(Gexpress~Line*Infect+(1|ID))

> m3<-lm(Gexpress~Line*Infect)

> m4<-lm(Gexpress~Line+Infect)

> anova(m1,m2,m3,m4)

refitting model(s) with ML (instead of REML)

Data: NULL

Models:

m4: Gexpress ~ Line + Infect

m1: Gexpress ~ Line + Infect + (1 | ID)

m3: Gexpress ~ Line * Infect

m2: Gexpress ~ Line * Infect + (1 | ID)

Df AIC BIC logLik deviance Chisq Chi Df Pr(>Chisq)

m4 4 1047.4 1063.7 -519.72 1039.4

m1 5 1045.6 1065.9 -517.80 1035.6 3.8410 1 0.05001 .

m3 5 1047.8 1068.2 -518.90 1037.8 0.0000 0 1.00000

m2 6 1046.4 1070.8 -517.22 1034.4 3.3645 1 0.06662 .

---

Signif. codes: 0 ‘***’ 0.001 ‘**’ 0.01 ‘*’ 0.05 ‘.’ 0.1 ‘ ’ 1

**(b)**

> m1<-lmer(Gexpress~Line*Infect+(1|ID))

> m2<-lmer(Gexpress~Line+Infect+(1|ID))

> m3<-lm(Gexpress~Line+Infect)

> m4<-lm(Gexpress~Line*Infect)

> anova(m1,m2,m3,m4)

refitting model(s) with ML (instead of REML)

Data: NULL

Models:

m3: Gexpress ~ Line + Infect

m2: Gexpress ~ Line + Infect + (1 | ID)

m4: Gexpress ~ Line * Infect

m1: Gexpress ~ Line * Infect + (1 | ID)

Df AIC BIC logLik deviance Chisq Chi Df Pr(>Chisq)

m4 4 511.08 523.60 -251.54 503.08

m1 5 513.08 528.73 -251.54 503.08 0.0000 1 1

m3 5 511.97 527.62 -250.99 501.97 1.1084 0 <2e-16 ***

m2 6 513.97 532.75 -250.99 501.97 0.0000 1 1

---

Signif. codes: 0 ‘***’ 0.001 ‘**’ 0.01 ‘*’ 0.05 ‘.’ 0.1 ‘ ’ 1
